# Ruptured coronary sinus aneurysm in pregnancy combined with cardiogenic shock and elevated V1 and aVR ST segments: Case report

**DOI:** 10.1097/MD.0000000000038788

**Published:** 2024-09-06

**Authors:** Yanhui Sun, Xing Lu, Honglan Ma

**Affiliations:** a Department of Neurology, The First Affiliated Hospital of Xi’an Medical University, Xi’an, Shaanxi Province, China; b Xi’an Medical University, Xi’an, Shaanxi Province, China; c Department of Respiratory and Critical Care Medicine, The First Affiliated Hospital of Xi’an Medical University, Xi’an, Shaanxi Province, China; d Department of Cardiovascular Medicine, The First Affiliated Hospital of Xi’an Medical University, Xi’an, Shaanxi Province, China.

**Keywords:** acute left coronary artery trunk occlusion, acute myocardial infarction, pregnancy female, rupture, sinus of Valsalva aneurysm (SOVA)

## Abstract

**Introduction:**

Sinus of Valsalva aneurysm (SOVA), a rare cardiac malformation, is usually congenital and rarely acquired and most commonly occurring in the right coronary sinus. The clinical presentation of patients with SOVA varies. It is usually asymptomatic when it has not ruptured, and when it compresses neighboring structures or ruptures, it can lead to heart failure or shock, at which point urgent surgical intervention is usually required. Rupture of the sinus of Valsalva aneurysm (RSOVA) during pregnancy is really hard to come by, especially if the clinical presentations resemble that of an acute myocardial infarction. This report describes a pregnant woman with severe chest pain and hypotension with aVR and V1 ST-segment elevation due to RSOVA.

**Patient concerns:**

Effects of RSOVA on the fetus, disease survival, and prognosis.

**Diagnosis:**

RSOVA.

**Interventions:**

Open SOVA repair.

**Outcomes:**

The patient’s blood pressure returned to normal range and clinical symptoms disappeared after the surgery. After 3 months of follow-up, the patient was hemodynamically stable without chest discomfort, and an echocardiogram showed a normal aortic sinus.

**Conclusion:**

Progressive aneurysm dilatation or rupture has a poor prognosis. A thorough history and physical examination are fundamental, with echocardiography being the initial diagnostic tool of choice, and other ancillary tests (e.g., computed tomography) being used to complement and confirm the diagnosis. Surgery remains the current treatment of choice for patients with RSOVA, while the continuation of pregnancy in pregnant patients with RSOVA remains a case-by-case measure.

## 1. Introduction

Sinus of Valsalva aneurysm (SOVA), a rare cardiac malformation, is usually congenital and rarely acquired and most commonly occurring in the right coronary sinus. The clinical presentation of patients with SOVA varies. It is usually asymptomatic when it has not ruptured, and when it compresses neighboring structures or ruptures, it can lead to heart failure or shock, at which point urgent surgical intervention is usually required. Rupture of the sinus of Valsalva aneurysm (RSOVA) during pregnancy is really hard to come by, especially if the clinical presentations resemble that of an acute myocardial infarction. This report describes a pregnant woman with severe chest pain and hypotension with aVR and V1 ST-segment elevation due to RSOVA. The study followed SCARE guidelines^[1]^ and informed consent was obtained from the patients.

## 2. Case report

We describe the case of RSOVA with chest pain and hypotension in a 27-year-old female with early pregnancy. Acute coronary syndrome (left main stem occlusion) was considered at the beginning with significant elevation of ultrasensitive troponin, ST-segment depression in electrocardiography (ECG) leads I, II, avF, avL, V2–V6, and ST-segment elevation in leads V1 and aVR. However, emergency coronary angiography showed normal vessels and extracorporeal pulmonary oxygenation support therapy was performed to maintain the patient’s circulation, followed by cardiac ultrasound and emergency surgery to confirm SOVA and successful SOVA repair was performed.

A 27-year-old female in her third month of pregnancy with a negative history of any particular diseases and family presented to the emergency room, with excruciating chest pain without obvious cause. Her chest pain persisted without relief, accompanied by chest tightness, shortness of breath, panic, nausea, and vomiting 3 times, and the vomit was stomach contents without blood. We performed a careful physical examination, which suggested tachycardia (heart rate: 110 beats/min) and profound shock (blood pressure [BP]: 58/30 mm Hg).

**Figure 1.**
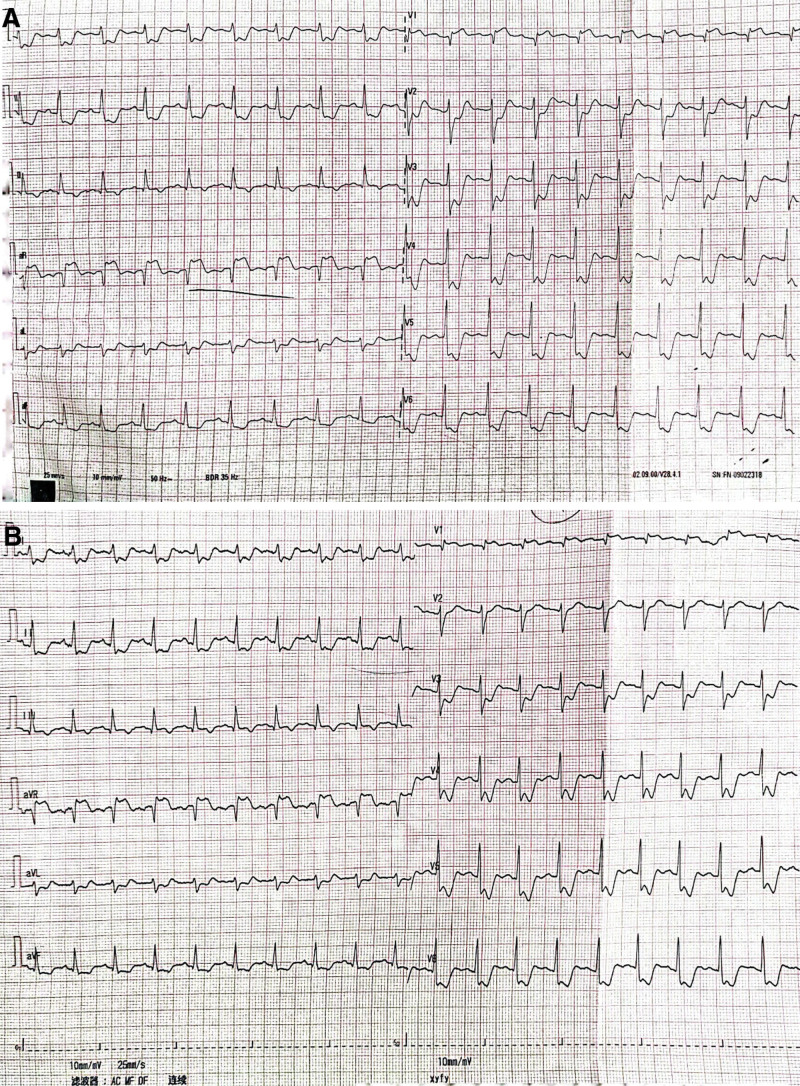
(A) Initial 12-lead electrocardiography revealed ST-segment elevation in the V1 and aVR leads with V2–V6, leads I, II, avF, and avL ST depressions. Note the ST-segment elevation in the aVR lead is more prominent than in the V1 lead. (B) A 12-lead electrocardiography reviewed half an hour later shows ST-segment elevation in leads V1 and aVR and ST-segment depression in leads V2 through V6, I, II, avF, and avL.

Emergency ECG suggested ST-segment depression in leads I, II, avF, avL, and V2–V6 and ST-segment elevation in leads V1 and aVR (see Fig. 1A, in which initial 12-lead ECG revealed ST-segment elevation in the V1 and aVR leads with V2–V6, lead I, II, avF, and avL ST depressions. Note the ST-segment elevation in the aVR lead is more prominent than in the V1 lead; and Fig. 1B, in which a 12-lead ECG reviewed half an hour later shows ST-segment elevation in leads V1 and aVR and ST-segment depression in leads V2 through V6, I, II, avF, and avL). Ultrasensitive troponin was 0.479 ng/mL. Combined with the ECG ST-segment changes and hypersensitive troponin, we immediately thought of the left main stem occlusion acute coronary syndrome. In view of the high risk of sudden death from left main stem occlusion, the coronary intervention was performed after a full discussion with the patient and her family. We deeply regret that we had to give up the unborn child to protect the mother’s safety.

**Figure 2.**
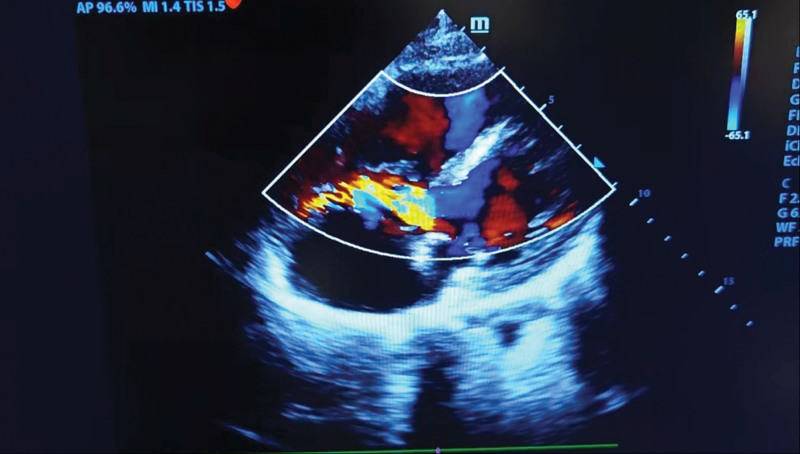
Transesophageal echocardiography with color Doppler showing a high-velocity multicolored (aliasing) mosaic of blood flow through the base of the sinus tumor.

After double antiplatelet therapy with aspirin and ticagrelor, coronary artery angiography was completed in an emergency. However, the angiography results showed no stenosis and thrombus in both left and right coronary arteries with Thrombolysis in Myocardial Infarction grade 3 in the distal flow. We then performed aortic arch angiography and found no dissection or stenosis. The situation was urgent, so the patient was treated with continuous extracorporeal pulmonary oxygenation support after communication and consultation with the patient’s family. At the same time, the bedside transthoracic echocardiography (TTE) was performed and revealed a continuous left-to-right sinus shunt, a large right heart system, reduced short-axis shortening (FS) of the left ventricle (26%), normal left ventricular systolic function, and no segmental wall motion abnormalities or ventricular chamber enlargement, suggesting a possible rupture of the right aortic sinus aneurysm (see Fig. 2, in which transesophageal echocardiography with color Doppler showing a high-velocity multicolored [aliasing] mosaic of blood flow through the base of the sinus tumor; and Video, Supplemental Digital Content, http://links.lww.com/MD/N272, in which echo loss was seen in the right coronary sinus of the aorta, with slightly enhanced echoes in the dissection, approximately 10 mm, and colorful left-to-right blood flow could be seen passing through).

These findings suggested rupture of the right coronary sinus SOVA and the patient was in a state of persistent severe shock. Therefore, after comprehensive consideration, emergency surgical exploration was performed to confirm the SOVA and successfully repaired the right RSOVA. Finally, the patient’s BP gradually returned to normal range and clinical symptoms disappeared after the surgery. After 3 days of observation and treatment in the intensive care unit, she was transferred to the general ward with stable vital signs. The patient was discharged from the hospital with no residual symptoms on the 13th postoperative day. As the patient returned to her place of residence, which was far away from our hospital, the patient was followed up in the local hospital. After 3 months of follow-up, the patient was hemodynamically stable without chest discomfort, and an echocardiogram showed a normal aortic sinus. The patient was very satisfied due to the clinically asymptomatic and stable BP.

This case report was approved by the patient and institutional review board.

## 3. Discussion

SOVA is a rare cardiac malformation, usually congenital and rarely acquired, most commonly occurring in the right coronary sinus (80.7%), followed by the noncoronary sinuses (15.8%), and rarely in the left sinus (3.5%).^[2]^ SOVAs can be congenital or acquired.^[3]^ Congenital aneurysms are underlying local weakness of the elastic membrane at the junction of the aortic mesentery and the fibrous ring.^[3]^ Typically, SOVA is more common in males than in females and is particularly prevalent in Asian countries.^[4]^ RSOVA requires early surgical intervention such as 1-stage suturing, patch repair, or aortic root replacement (with or without valve replacement), and if left untreated, patients have a median survival of 3.9 years.^[5,6]^

We present a female patient with RSOVA resembling a left main coronary artery occlusion in early pregnancy, and 2 aspects of this case are of particular interest. First, patients with SOVA who present with typical left main coronary artery occlusion as their first presentation may be difficult to diagnose because of its strong resemblance to acute myocardial infarction. The emergency ECG in this case showed ST-segment depression in leads I, II, avF, avL, and V2–V6 and ST-segment elevation in leads V1 and aVR. Acute chest pain with ST-segment elevation in the aVR lead and ST-segment depression in several other leads can indicate acute left main coronary artery obstruction.^[7]^ The ECG lead aVR is used to obtain specific information about the right side of the heart such as the right ventricular outflow tract (RVOT) and the base of the septum. RSOVA can cause acute interruption of coronary blood flow with continuous flow into the right ventricle (RV) and acute left-to-right shunting leading to inadequate coronary blood supply, which can be severe enough to result inlead to cardiogenic shock, and then exacerbate coronary artery insufficiency, and lead to ST-segment elevation in the aVR and diffuse ST-segment depression. This may be related to acute RV injury induced by sympathetic overexcitation inducing RV transmural ischemia.^[8,9]^ Therefore, the patient’s presentation is very similar to that of occlusion of the left main coronary artery. In our clinical practice, we need to recognize RSOVA in time to distinguish it from acute myocardial infarction by careful physical examination and echocardiography. SOVA was not suspected in time after coronary angiography was unremarkable, and a continuous murmur at the left edge of the sternum was recorded and described for the first time in the cardiac care unit. Refinement of bedside echocardiography suggests SOVA. Appropriate use of noninvasive tests such as echocardiographic evaluation in patients with acute cardiovascular disease can help in differential diagnosis and avoid unnecessary medical interventions.

Before the introduction of echocardiography, the diagnosis of RSOVA in a living patient was rare, so most reports came from autopsy or surgery. Today, TTE often aids in diagnosis and provides very detailed information to the clinician. TTE or transesophageal echocardiography allows accurate determination of aneurysm size, sinus of origin, communicating chamber, degree of valve regurgitation, and associated anomalies.^[10,11]^ Chu et al^[12]^ showed that aortography could show aortic regurgitation in some SOVA patients, but not in our patient. RSOVA may result in acute hemodynamic instability; therefore, early recognition is important and often requires surgical intervention.^[13]^ Echocardiography, ECG-gated cardiac computed tomography angiography, and cardiac magnetic resonance imaging can provide accurate diagnosis.^[9]^ A detailed symptom-oriented, targeted physical examination in the emergency room reveals a distinct continuous murmur at the left sternal margin.

Second, the patient’s pregnancy was combined with RSOVA, which is rare in clinical reports,^[4]^ and long-term extensive follow-up trials are lacking. Chen et al^[4]^ reported a case of a 26-year-old woman diagnosed with a ruptured SOVA into the RVOT at 18 weeks’ gestation (asymptomatic), who delivered a healthy baby boy by cesarean section after an uncomplicated full-term gestation; surgery was performed on the 50th day postpartum to ruptured aneurysm repair was completed. Fang et al^[14]^ reported 2 patients with RSOVA (presenting with a sore throat, cough, fever, dizziness, dyspnea, palpitations, and lower abdominal distension, respectively) who were delivered by cesarean section after epidural anesthesia, and underwent surgical correction of the SOVA and ventricular septal defect on 13 and 7 days postoperatively, respectively. Agrawal et al^[2]^ reported for the first time a case of a healthy fetus delivered by elective cesarean section after transcatheter occlusion in a female patient with RSOVA (mild dyspnea) at 26 weeks of gestation. Our patient developed hemodynamic instability and eventually had surgery to complete the SOVA repair and unfortunately, the fetus was miscarried. Early reports of cardiopulmonary bypass and valve replacement in pregnancy are associated with 16% to 33% fetal mortality.^[15]^ If the patient’s condition permits, cardiac surgery is best avoided in early pregnancy and can be performed after the 13th week of pregnancy, taking into account the safety of the fetus. However, if the patient is critically ill or exhibits obvious symptoms associated with RSOVA, such as heart failure, immediate surgical intervention should be performed and the pregnancy can then be terminated.^[15]^

During pregnancy, the cardiovascular system is progressively stressed due to increased blood volume, increased heart rate, and increased output per beat, which can be very dangerous for pregnant women with underlying heart conditions.^[14]^ Hemodynamic changes during delivery can exacerbate aneurysms, so cesarean section is the preferred option for pregnant women with RSOVA.^[4]^ However, there is currently no consensus on the timing of surgical correction of SOVA. Cesarean section is appropriate before repair of RSOVA near term, however, if it occurs early in pregnancy, the decision to terminate the pregnancy will involve balancing the risk of early delivery of the fetus with the hemodynamic consequences for the mother of continuing the pregnancy.^[14,16]^ Therefore, the optimal time for surgical repair should be determined by the patient’s specific condition. If the patient is critically ill or develops significant symptoms associated with RSOVA, surgery should be performed immediately.^[4]^ To avoid the risk of fetal abortion, transcatheter closure of an RSOVA may be more appropriate for pregnant women than traditional open-heart surgery, but surgical treatment is still currently preferred.^[2]^ General anesthesia and epidural anesthesia can also be used safely in women with ruptured aortic sinus aneurysms.^[4,14]^

Congenital SOVA is often associated with Marfan syndrome, Ehlers–Danlos syndrome, or other connective tissue disorders. Acquired aneurysms are caused by diseases affecting the aortic wall, such as infections (syphilis, bacterial endocarditis, or tuberculosis), trauma, or connective tissue disease.^[17–19]^ Takach et al^[20]^ showed that the rupture occurred more frequently in the right SOVA. Most ruptured aortic valves entered the RV (60%) or right atrium (29%); only a few ruptured into the left atrium (6%), left ventricle (4%), or pericardium (1%). The clinical features of patients with SOVA range from asymptomatic to heart failure. In 60% of patients, the progression of symptoms is insidious, making it impossible to date the RSOVA.^[12]^ RSOVA can be accompanied by dyspnea, palpitations, fatigue, syncope, chest pain, or decreased exercise tolerance, and causes acute symptoms in only about one-third of previously healthy patients (like ours).^[11,19]^ The most common sign of RSOVA is a loud machine-like systolic and diastolic murmur with strong atrial fibrillation at the left border of the sternum, which suggests a rupture of the right coronary sinus aneurysm into the RVOT.^[12]^ It can be detected by a careful clinical examination. Therefore, be alert to the possibility of RSOVA when acute hemodynamic disturbances with a distinctive left sternal border continuity murmur are encountered in the clinic.

In conclusion, RSOVA can present as a clinical emergency and lead to progressive worsening dyspnea and acute coronary syndrome. A persistent heart murmur with acute shock suggests acute RSOVA.^[9]^ All acute cardiovascular conditions should be evaluated using echocardiography, including cardiogenic shock and other different causes of shock, chest trauma, acute myocardial infarction and ischemia, acute pulmonary embolism, pericardial tamponade, and aortic coarctation, to clarify the cause of acute symptoms based on the range of signs and symptoms.^[21]^

## 4. Conclusion

RSOVA in a pregnant woman is rare and may be asymptomatic before acute rupture; a thorough history and physical examination are fundamental. Echocardiography is the initial diagnostic tool of choice, and other imaging techniques such as cardiac angiography, computed tomography, and magnetic resonance imaging may be used to supplement or confirm the diagnosis. Progressive aneurysm dilatation or rupture has a poor prognosis.^[16]^ Despite the increasing use of transcatheter occlusion techniques, surgical repair remains the treatment of choice and carries a low surgical risk. Early in pregnancy, the timing of surgery still varies from case to case, and the decision to allow continuation of pregnancy depends on the balance between the risk of preterm labor to the child and the hemodynamic consequences of continuing the pregnancy later in life for the mother.^[16]^

## Acknowledgements

We are grateful to our patient and her family.

## Author contributions

**Conceptualization:** Yanhui Sun, Honglan Ma.

**Investigation:** Yanhui Sun, Honglan Ma.

**Writing—original draft:** Yanhui Sun.

**Writing—review and editing:** Yanhui Sun, Honglan Ma.

**Data curation:** Xing Lu.

**Methodology:** Honglan Ma.

**Supervision:** Honglan Ma.

## Supplementary Material


